# Supplementary material for: Effect of Exogenous Melatonin Application on the Grain Yield and Antioxidant Capacity in Aromatic Rice under Combined Lead–Cadmium Stress
Source: Antioxidants (Basel). 2022 Apr 13;11(4):776. doi: 10.3390/antiox11040776 (PMC9028010; doi:10.3390/antiox11040776)
Supplement: Supplementary file 1 [file antioxidants-11-00776-s001.zip › Supplementary Word S1.pdf]

Supplementary Word S1. Primary metabolite profiling

The sample extracts were analyzed using an UPLC-ESI-MS/MS system (UPLC, SHIMADZU Nexera X2, https://www.shimadzu.com.cn/; MS, Applied Biosystems 4500 Q TRAP, https://www.thermofisher.cn/cn/zh/home/brands/applied-biosystems.html). The analytical conditions were as follows, UPLC: column, Agilent SB-C18 (1.8 µm, 2.1 mm * 100 mm); The mobile phase was consisted of solvent A, pure water with 0.1% formic acid, and solvent B, acetonitrile with 0.1% formic acid. Sample measurements were performed with a gradient program that employed the starting conditions of 95% A, 5% B. Within 9 min, a linear gradient to 5% A, 95% B was programmed, and a composition of 5% A, 95% B was kept for 1 min. Subsequently, a composition of 95% A, 5.0% B was adjusted within 1.1 min and kept for 2.9 min. The flow velocity was set as 0.35 mL per minute; The column oven was set to 40°C; The injection volume was 4 µL. The effluent was alternatively connected to an ESI-triple quadrupole-linear ion trap (QTRAP)-MS.

LIT and triple quadrupole (QQQ) scans were acquired on a triple quadrupole-linear ion trap mass spectrometer (Q TRAP), AB4500 Q TRAP UPLC/MS/MS System, equipped with an ESI Turbo Ion-Spray interface, operating in positive and negative ion mode and controlled by Analyst 1.6.3 software (AB Sciex). The ESI source operation parameters were as follows: ion source, turbo spray; source temperature 550°C; ion spray voltage (IS) 5500 V (positive ion mode)/-4500 V (negative ion mode); ion source gas I (GSI), gas II(GSII), curtain gas (CUR) were set at 50, 60, and 25.0 psi, respectively; the collision-activated dissociation(CAD) was high. Instrument tuning and mass calibration were performed with 10 and 100 µmol/L polypropylene glycol solutions in QQQ and LIT modes, respectively. QQQ scans were acquired as multiple reaction monitoring (MRM) experiments with collision gas (nitrogen) set to medium. DP and CE for individual MRM transitions was done with further DP and CE optimization. A specific set of MRM transitions were monitored for each period according to the metabolites eluted within this period. MRM scanning mode is used to identify metabolites in the samples in high-efficiency batch, so as to obtain more complete and accurate metabolic spectrum information. In the MRM mode, the quadrupole first filters the precursor ions (precursor ions) of the target substance, and excludes ions corresponding to other molecular weight substances to initially eliminate interference; the precursor ions are fragmented after the induced ionization of the collision chamber to form many fragment ions, fragment ions Then select a characteristic fragment ion by triple quadrupole filtering to eliminate the interference of non-target ions, so that the quantification is more accurate and the repeatability is better. After obtaining the metabolite mass spectrometry data of different samples, the peak area of all substance mass peaks was integrated, and the peaks of the same metabolite in different samples were integrated and corrected.

The data was analysed using the following methods:

1) KEGG annotation and enrichment analysis: Identified metabolites were annotated using KEGG Compound database (http://www.kegg.jp/kegg/compound/), annotated metabolites were then mapped to KEGG Pathway database (http://www.kegg.jp/kegg/pathway.html). Pathways with significantly regulated metabolites mapped to were then fed into MSEA (metabolite sets enrichment analysis), their significance was determined by hypergeometric test's p-values.

2) PCA: Unsupervised PCA (principal component analysis) was performed by statistics function prcomp within R (www.r-project.org). The data was unit variance scaled before unsupervised PCA. PLS-DA performs a log2 transformation on the raw data, followed by centering (Mean Centering), using the MetaboAnalystR package PLSR.Anal function in R software.

3) Differential metabolites: Significantly regulated metabolites between groups were determined by VIP ≥ 1 and absolute log2FC (fold change) ≥ 1. VIP values were extracted from OPLS-DA result, which also contain score plots and permutation plots, was generated using R package MetaboAnalystR. The data was log transform (log2) and mean centering before OPLS-DA. In order to avoid overfitting, a permutation test (200 permutations) was performed. Both Venn diagram and Bar chart were carried out by R package pheatmap.
